# Suprachoroidal Triamcinolone Plus Intravitreal Bevacizumab Versus Bevacizumab Alone for Diabetic Macular Edema in Pseudophakic Eyes: A randomized controlled trial

**DOI:** 10.12669/pjms.42.6.14576

**Published:** 2026-06

**Authors:** Imran Ahmad, Yousaf Jamal Mahsood

**Affiliations:** 1 Imran Ahmad, (MBBS, MRCS.Ed, FICO, FACS, FCPS, FCPS-Vitreoretina, MHPE) Associate Professor, Department of Ophthalmology, Khyber Medical College, Khyber Teaching Hospital, Peshawar, Pakistan; 2 Yousaf Jamal Mahsood, (MBBS, MHR, MRCSEd, FICO, FRCS, FCPS), Associate Professor, Department of Ophthalmology, Khyber Girls Medical College, Hayatabad Medical Complex, Peshawar, Pakistan

**Keywords:** Bevacizumab, Diabetic macular edema, Intravitreal injections, Suprachoroidal, Triamcinolone acetonide

## Abstract

**Objective:**

To determine the efficacy of intravitreal bevacizumab (IVB) monotherapy versus a combination of IVB and suprachoroidal triamcinolone acetonide (SCTA) followed by monthly IVB in pseudophakic eyes with center-involving diabetic macular edema (DME).

**Methodology:**

The Ophthalmology Department of Khyber Teaching Hospital conducted this randomized controlled study between April 2023 to February 2024. Eighty-two pseudophakic patients with center-involving DME were randomly divided into two groups (41 each). The monotherapy group (Group-I) received a monthly injection of IVB (1.25mg/0.05 mL) for three months. The combination therapy group (Group-II) received combination of IVB (1.25 mg/0.05 mL) and SCTA (4 mg/0.1 mL) injections at first visit which were then followed by monthly IVB injection for next two months. Best-corrected visual acuity (BCVA) and central macular thickness (CMT) were measured at baseline and at four months.

**Results:**

Both groups showed statistically significant improvement in BCVA and CMT from baselines. In Group-I, BCVA improved from 0.90±0.31 to 0.69±0.31 (p<0.001), and CMT decreased from 385.12±68.51 µm to 329.22±55.70 µm (p<0.001). In Group-II, BCVA improved from 0.84±0.28 to 0.52±0.21 (p<0.001), while CMT decreased from 382.49±63.32 µm to 292.46±32.24 µm (p<0.001). When compared between the groups, Group-II showed statistically significant improvement both in BCVA (p=0.034) and CMT (p<0.001).

**Conclusion:**

In pseudophakic patients with center-involving DME, a single combined treatment with SCTA and IVB yields better anatomical response and visual improvements as compared to IVB monotherapy. This combination approach might provide more therapeutic efficacy in terms of reducing macular edema.

***RCT Registration No.:*** ClinicalTrials.gov. (NCT06882551).

## INTRODUCTION

Diabetes is becoming more prevalent worldwide, and it is predicted that over 600 million individuals will have the disease by 2040.^1^ Pakistan’s diabetes prevalence has dangerously increased from 11.7% in 2016 to 26.7% in 2022, placing it third in the world behind China and India.^2^ The number of people at risk for diabetic retinopathy (DR) is rising along with this exponential increase. Diabetic macular edema (DME) continues to be the most prevalent cause of visual impairment in people of working age among the different sight-threatening symptoms of DR.^3^

There are several approaches to manage DME. The Early Treatment Diabetic Retinopathy Study (ETDRS) established focal/grid laser photocoagulation as the gold standard for treating clinically significant macular edema.^4^ However, focused laser has become less common with the introduction and widespread use of intravitreal anti-vascular endothelial growth factor (anti-VEGF) therapy, and anti-VEGF drugs are currently considered as first-line therapy for the majority of DME patients.^5^ Although anti-VEGF therapy may restore macular architecture and improve visual acuity, about 25% of patients do not have significant functional improvement.^6^ The multifactorial pathophysiology of DME, in which oxidative stress from long-term hyperglycemia causes persistent inflammation and the production of inflammatory mediators—processes that are only partially responsive to anti-VEGF therapy—is a key explanation.^7^

Intravitreal corticosteroids were commonly used before the anti-VEGF era, and there is plenty of evidence to support their utility in treating DME.^7^ They are still an appropriate option in many situations, especially when anti-VEGF is inaccessible, contraindicated, or in pseudophakic eyes. Steroids directly target pathways not addressed by VEGF blocking alone by reducing vascular permeability and exerting profound anti-inflammatory and anti-angiogenic effects.^8^ In contrast to intravitreal administration, suprachoroidal injection of triamcinolone acetonide has recently gained popularity as a delivery method that allows for high drug concentration in the retina and choroid, with less anterior region exposure and subsequently fewer risks of glaucoma and cataract.^9^,^10^ In retinal vascular disorders such central retinal vein blockage, combination therapy using intravitreal bevacizumab and suprachoroidal triamcinolone acetonide has demonstrated encouraging morphological improvements.^11^

Nevertheless, there is still a dearth of information regarding this combination strategy in pseudophakic DME. Although triamcinolone has been studied in pseudophakic DME, either by itself or in conjunction with laser, there is currently little meaningful data for comparison with regular bevacizumab monotherapy. Based on the aforementioned evidence, we hypothesize that in pseudophakic DME eyes, the initial addition of suprachoroidal triamcinolone acetonide to intravitreal bevacizumab will lead to a higher reduction of macular edema and better functional visual results than bevacizumab monotherapy. We believe that combination therapy will produce a more robust and long-lasting therapeutic response by concurrently addressing inflammation-mediated vascular permeability and VEGF-driven angiogenesis.

Thus, the specific clinical question is still unanswered: does intravitreal bevacizumab plus a single initial suprachoroidal triamcinolone acetonide injection produce better anatomical and visual results in pseudophakic DME patients than bevacizumab monotherapy alone? This is why we designed this study to compare the effectiveness of combination therapy (initial intravitreal bevacizumab plus suprachoroidal triamcinolone acetonide followed by additional monthly bevacizumab injections for two months) versus monotherapy with monthly intravitreal bevacizumab injection for three months in pseudophakic patients with DME. If the combined regimen demonstrates superior outcomes, this could justify modification of current treatment protocols especially in pseudophakic eyes — by incorporating suprachoroidal steroid delivery to achieve faster and more pronounced anatomical recovery and improved visual outcomes in DME.

## METHODOLOGY

This randomized controlled study was carried out in the Department of Ophthalmology, Khyber Teaching Hospital, Peshawar, from April 2023 to February 2024.

### Ethical Approval:

The approval was obtained from the Institutional Ethical and Review Board (IERB) (Notification No: 450/DME/KMC; dated: August 3, 2023).

The trial (NCT06882551) was prospectively registered on ClinicalTrials.gov. Based on the results of a previous study reporting a mean central macular thickness of 377 ± 117 µm after IVB,^12^ and assuming a clinically meaningful 20% reduction (75.4 µm), the required sample size was calculated using G*Power to be 30 eyes per group, using 80% power and a one-sided α of 0.05. After accounting for a 25% anticipated dropout rate, the final sample size was increased to 41 eyes per group.

### Inclusion Criteria:

Patients who fulfilled inclusion criteria were enrolled consecutively until the desired sample size was achieved. Inclusion criteria were pseudophakic patients with any stage of Non-Proliferative Diabetic Retinopathy (NPDR) with center-involved DME, which was characterized by involvement of the central 1000-µm subfield of the ETDRS grid on spectral-domain OCT (Heidelberg OCT II) with a central macular thickness (CMT) greater than 250 µm.

### Exclusion Criteria:

Glaucoma or intraocular pressure greater than 21 mmHg, phakic eyes, corneal opacity, vitreous hemorrhage, proliferative diabetic retinopathy, vitreomacular traction, epiretinal membrane, previous anti-VEGF treatment, or a history of pan retinal photocoagulation were excluded.

Two equal groups (41 in each) were randomly selected from among the participants using the lottery method. Each participant provided written informed consent, and coded electronic identifiers were used to conceal patient identification. The investigators involved in the measurement of CMT and BCVA of our study participants were masked.

All participants underwent a comprehensive ophthalmic examination at baseline including best-corrected visual acuity (BCVA) measured on logMAR, anterior segment assessment with intraocular pressure measurement, dilated fundus evaluation, and OCT-based measurement of CMT. In the monotherapy group (Group-I), the participants received monthly intravitreal injection of bevacizumab (1.25 mg/0.05 mL) for three months. In the combination therapy group (Group-II), one suprachoroidal injection of triamcinolone acetonide (4 mg/0.1 mL) combined with intravitreal bevacizumab (1.25 mg/0.05 mL) was administered within one week of suprachoroidal injection followed by monthly intravitreal bevacizumab injection (1.25 mg/0.05 mL) for next two months. At four months, BCVA (logMAR) and CMT were reassessed, and the change from baseline was compared between groups. There were no significant complications encountered except transient rise in intraocular pressure (IOP) and pain after suprachoroidal injection relieved by systemic analgesics and carbonic anhydrase inhibitors. No complication was documented on follow up visits.

### Statistical analysis:

It was performed using SPSS version 26. Quantitative variables were expressed as mean and standard deviation, whereas categorical variables were expressed as frequencies and percentages. Independent sample t-test was applied for inter-group comparisons of means while paired sample t-test was applied for comparison of means within the group. A p-value of less than 0.05 was considered statistically significant.

## RESULTS

**Fig.1:**
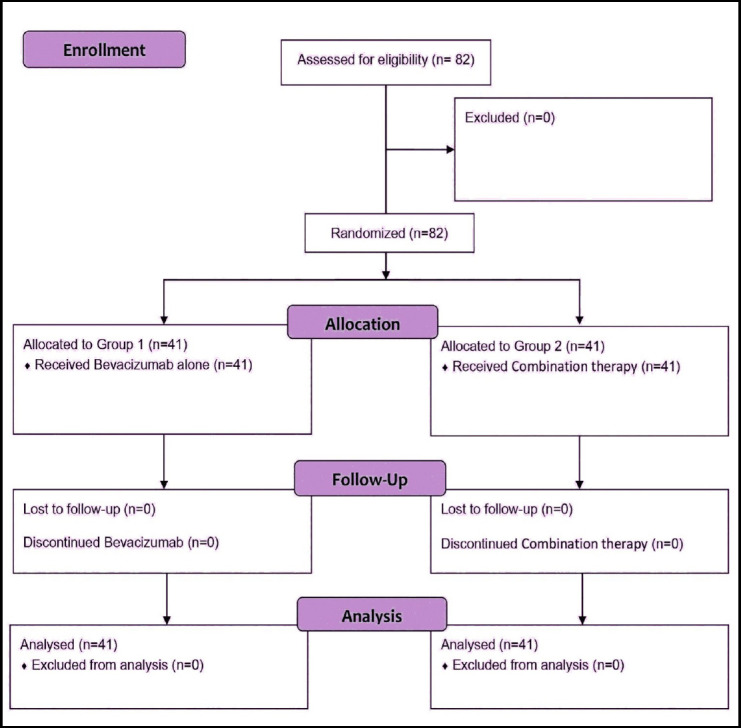
CONSORT 2010 Flow Diagram.

A four-month follow-up was completed by 82 pseudophakic eyes (41 in each group) with center-involved DME. The CONSORT 2010 flow diagram is shown in Fig.1. The study group’s mean age was 56.49+7 years, with 43 (52.4%) males. The monotherapy (Group-I) and combination therapy (Group-II) did not differ statistically significantly with regard to baseline demographics, visual acuity, or CMT (Table-I). From baseline to four months, both groups showed significant within-group improvements in BCVA and decreases in CMT (all p<0.001) (Table-II).

**Table-I:** Comparison of the demographics at baseline.

Parameter, (N=82)		Group-I	Group-II	p-value
Gender, n (%)	Male	22 (51.2)	21 (48.8)	0.83^1^
	Female	19 (48.7)	20 (51.3)	
Age in years, (mean + SD)		56.44 + 7.35	56.54 + 6.73	0.95^2^
Pre-treatment VA in LogMAR, (mean + SD)		0.9 + 0.31	0.84 + 0.28	0.33^2^
Pre-treatment CMT in microns, (mean + SD)		385.12 + 68.51	382.49 + 63.32	0.86^2^

N=total sample, n=frequency, %=percentage, SD=standard deviation, VA-visual acuity, LogMAR=logarithm of miminal angle of resolution, CMT=central macular thickness. Group-I= Bevacizumab alone, Group-II=Combination therapy.

1 Chi-square test was applied.

2 Independent sample t-test was applied.

**Table-II:** Comparison within the groups at the study endpoint.

Parameter, (N=82)		Pre-treatment	Post-treatment	95% Confidence interval		p-value*
				Lower	Upper	
Group-I	VA in LogMAR, (mean + SD)	0.9 + 0.31	0.64 + 0.31	0.19	0.32	<0.001
	CMT in microns, (mean + SD)	385.12 + 68.51	329.22+ 55.7	42.28	69.53	<0.001
Group-II	VA in LogMAR, (mean + SD)	0.84 + 0.28	0.52 + 0.21	0.26	0.37	<0.001
	CMT in microns, (mean + SD)	382.49 + 63.32	292.46 + 32.24	72.81	107.24	<0.001

N=total sample, SD=standard deviation, VA-visual acuity, LogMAR=logarithm of miminal angle of resolution, CMT=central macular thickness. Group-I= Bevacizumab alone, Group-II= Combination therapy.

* Paired sample t-test was applied.

The combination therapy group outperformed the monotherapy group in terms of visual acuity at the study’s endpoint (0.52±0.21 vs. 0.64±0.31; p=0.034). Table-III illustrates that the combination therapy group’s central macular thickness was substantially lower than that of the monotherapy group (292.46±32.24 µm vs. 329.22±55.70 µm; p<0.001). Overall, at four months, treatment with intravitreal bevacizumab with initial suprachoroidal triamcinolone acetonide demonstrated greater morphological and functional improvement than bevacizumab monotherapy.

**Table-III:** Comparison between the groups at the study endpoint.

Parameter, (N=82)	Group-I	Group-II	95% Confidence interval		p-value*
			Lower	Upper	
VA in LogMAR, (mean + SD)	0.64 + 0.31	0.52 + 0.21	0.01	0.24	0.034
CMT in microns, (mean + SD)	329.22 + 55.7	292.46 + 32.24	16.75	56.76	<0.001

N=total sample, SD=standard deviation, VA-visual acuity, LogMAR=logarithm of miminal angle of resolution, CMT=central macular thickness. Group-I= Bevacizumab alone, Group-II= Combination therapy.

* Independent sample t-test was applied.

## DISCUSSION

The anatomical response of anti-VEGF and suprachoroidal triamcinolone acetonide combined therapy was compared to anti-VEGF monotherapy in this study. Our results show that a combination therapy achieved a better central macular thickness (CMT) reduction. In particular, the combination arm’s mean CMT reduction was 90.03 microns, whereas the monotherapy groups were 55.90 microns. This difference was statistically significant (p = 0.001), with an absolute difference of 34.13 microns. The likely explanation of this difference could be that diabetic macular edema (DME) is a complex condition that is likely caused by both inflammatory and vascular routes and combination therapy may have addressed both components as compared to monotherapy.^13^ Anti-VEGF agents mainly target the VEGF-mediated pathway, although the vitreous of diabetes patients has higher levels of inflammatory mediators, such as cytokines, TNF-α, interleukins, and matrix metalloproteinases, which are linked to the disruption of the blood–retinal barrier and the development of edema.^14^,^15^ Anti-VEGF monotherapy therefore may have limited efficacy in patients where the inflammatory component is predominant.

Suprachoroidal triamcinolone acetonide has previously been demonstrated to be both safe and effective.^16^ Intravitreal steroids are at least as effective as anti-VEGF in enhancing visual acuity and decreasing edema in DME, according to the BEVORDEX trial.^17^ Despite being successful, intravitreal triamcinolone is linked to greater rates of intraocular pressure rise and cataract formation. Nevertheless, anti-VEGF continues to be the standard of treatment, supported by numerous landmark trials.^13^ Combining intravitreal bevacizumab with suprachoroidal triamcinolone has proven to improve visual results, minimize the amount of anti-VEGF injections needed, and diminish CMT in pseudophakic patients.

Although there was no significant difference in visual improvement, the TYBEE study (Barakat MR et al., Wykoff CC et al.) also found that the combination of suprachoroidal triamcinolone and aflibercept generated better anatomic results and required fewer aflibercept injections than monotherapy.^18^ The idea that ranibizumab plus a corticosteroid (dexamethasone implant) produces better anatomical results than ranibizumab alone is also supported by DRCR.net Protocol U.^19^

In order to precisely treat the inflammatory and VEGF-driven aspects of DME, we chose to combine suprachoroidal triamcinolone with intravitreal bevacizumab in our trial. This strategy makes sense because the predominance of inflammatory mediators causes some patients to react less favorably to anti-VEGF alone. The HULK study also demonstrated minimal anterior chamber levels of triamcinolone following suprachoroidal injection, supporting its safety with respect to anterior segment complications, and confirming its efficacy and tolerability in DME.^21^

### Limitations:

Our study’s randomized control design and investigator masking were its strongest points, but the inclusion solely of pseudophakic individuals was a limitation. Therefore, more research is required to assess how suprachoroidal triamcinolone affects the development or advancement of cataracts in phakic patients. Further research is necessary even though some evidence points to low lens exposure when using the suprachoroidal approach. The investigation was carried out at a single location; in order to improve generalizability, multicenter trials with bigger sample sizes are required. Further research is required to determine how other systemic factors affect DME response because we did not include patients with systemic comorbidities other than diabetes.

The brief follow-up is another limitation. To assess full remission, recurrence after the drug’s effects wear off, and the necessity of re-treatment, a longer follow-up is required. Furthermore, glycemic control (HbA1c), which is known to have a direct impact on DME progression and treatment response, was not evaluated. It is advised that future research examine the relationship between metabolic management and morphological and functional outcomes in individuals receiving combination therapy.

## CONCLUSION

For diabetic macular edema, a single injection of suprachoroidal triamcinolone acetonide combined with monthly intravitreal bevacizumab seems to be an effective treatment strategy that improves anatomical outcomes of central macular thickness and improved visual outcome.

### AI tool(s) Statement:

ChatGPT and Quill Bot was used for coherence and paraphrasing. After using these AI tools the author(s) reviewed and edited the content as needed and take(s) full responsibility for the content of the publication.

### Author’s Contribution:

**IA YJM:** Both authors contributed to the concept, design, literature search, data acquisition, manuscript preparation, manuscript editing, final approval of manuscript and agreed to be accountable.
